# Associations Between Family Income and Children’s Physical Fitness and Obesity in California, 2010–2012

**DOI:** 10.5888/pcd12.140392

**Published:** 2015-02-12

**Authors:** Yichen Jin, Jessica C. Jones-Smith

**Affiliations:** Author Affiliation: Yichen Jin, Johns Hopkins Bloomberg School of Public Health, Baltimore, Maryland.

## Abstract

**Introduction:**

Socioeconomic status may influence childhood obesity prevalence and children’s fitness level. The purpose of this study was to assess the association between family income and children’s physical fitness level and obesity prevalence for 8 racial/ethnic groups.

**Methods:**

Data for 1,617,400 fifth-, seventh-, and ninth-grade children who took a physical fitness test from 2010 through 2012 in California were used in this cross-sectional study. Multiple linear and log-binomial regressions were used to test whether low family income (as indicated by eligibility for National School Lunch Program) was associated with physical fitness level or obesity prevalence. Differences were tested by race/ethnicity while adjusting for age and sex. Fitness score was measured on a scale from 0 (least healthy) to 6 (most healthy).

**Results:**

Average fitness score was 4.45 (standard deviation, 1.47). Prevalence of obesity was 20.3%, and 56% of children were classified as having lower family income. Lower family income (vs higher) was associated with lower fitness score (coefficient = −0.57; 95% confidence interval [CI], −0.62 to −0.53). Lower-income children had higher prevalence of obesity (relative risk = 1.81; 95% CI, 1.72–1.89) compared with higher-income children. These inverse associations were seen among American Indian, Asian, Pacific Islander, Filipino, Hispanic/Latino, African American, and white children and among children who were identified as being of 2 or more races/ethnicities.

**Conclusion:**

Children with lower family incomes tend to have less healthy physical fitness status and have higher risk of obesity than children with higher family incomes. This information can be used to help set policies and provide programs aimed at improving fitness and decreasing obesity risk among low-income children.

## Introduction

Childhood obesity and physical inactivity are major public health concerns; 32% of US children are either overweight or obese, and only 37% of students meet the physical activity recommendations of at least 60 minutes on at least 5 days per week (1,2). Obese children have higher risk of obesity in adulthood (3), which is associated with comorbidities (4). Physical inactivity is an independent risk factor for obesity and cardiovascular disease among children (5).

Socioeconomic status (SES) may play a role in both childhood obesity prevalence and children’s physical fitness levels. Low SES can be a barrier to physical activity and healthful eating (6–8), and children who regularly perform high-intensity activity tend to be more physically fit (9). Previous studies have reported that children of low SES have higher risk of obesity than children of high SES (6,7). Fewer studies have examined the effect of SES on physical fitness. Although racial/ethnic disparities in obesity have been reported (1,10), few studies have examined the relationship between SES and obesity within racial/ethnic groups. Data from the US National Health and Nutrition Examination Survey (NHANES) indicated that SES is associated with lower risk for obesity only among white children (11). However, in that study, children with a range of ages (2–19 years) were grouped together; the sample size was only moderately sized after stratifying by race and sex; and only white, African American, and Hispanic populations were included.

We used data from children and adolescents from 8 racial/ethnic groups in California to assess the association between family income and 1) children’s physical fitness level, 2) children’s body mass index (BMI) *z*-score, and 3) childhood obesity. We hypothesized that children living in lower-income families would have lower physical fitness levels and higher risk for obesity and that the magnitude of the association may differ among racial/ethnic groups. On the basis of previous literature (1,10), we hypothesized that the associations would be stronger for boys than girls.

## Methods

### Study population and design

This cross-sectional study used physical fitness, anthropometric, and sociodemographic data obtained from the California Department of Education (12). Public and charter schools in California are required to administer a physical fitness test to all fifth-, seventh-, and ninth-grade students annually. The physical fitness test scores and basic demographic information are recorded for each child and submitted to the California Department of Education. Mandatory physical fitness testing began in 1999; however, information about children’s SES was only available for the 2010–2011 and 2011–2012 school years. Race or ethnicity was reported on school enrollment forms by parents, who were given the option to choose only 1 category from 8 groups.

There were 1,724,498 students with physical fitness test data from 2010 through 2012. Students who had missing information (National School Lunch Program [NSLP] status [n = 42,778], race/ethnicity [n = 8,099], BMI [n = 23,809], fitness score [n = 26,608]) were excluded. Additional exclusions included 72 students with an unclear or ineligible test date; 1,541 students with an implausible age (5th grade: <8 years or >13 years; 7th grade: <10 years or >15 years; 9th grade: <12 years or >17 years), and 4,191 students who had a BMI *z*-score, height *z*-score, or weight *z*-score higher than 5 or less than −5 (13,14). A total of 1,617,400 (93.8%) students made up the analytic sample.

### Dependent variables

#### Physical fitness assessment

The physical fitness test uses the Fitnessgram protocol (15) and assesses 6 fitness areas: 1) aerobic capacity, assessed by a 1-mile run or progressive aerobic cardiovascular endurance run or walk test (only for ages 13 or older); 2) abdominal strength and endurance, assessed by curl-ups; 3) upper body strength and endurance, assessed by push-ups, modified pull-ups, or flexed-arm hang; 4) body composition, assessed by skinfold measurements, BMI, or bioelectric impedance analyzer; 5) trunk extensor strength and flexibility, assessed by trunk lift; 6) and flexibility, assessed by back-saver sit-and-reach or shoulder stretch. Students receive 1 point if their test result falls in the Healthy Fitness Zone, the level associated with good health, in each fitness area (16). The total points from the 6 fitness areas are summed to provide the fitness score, ranging from 0 (least healthy) to 6 (most healthy).

#### BMI *z*-score and obesity

BMI *z*-scores were calculated using Stata version 12 (StataCorp, LP) using the 2000 Centers for Disease Control and Prevention (CDC) growth reference (17). Students were classified based on their BMI percentile as normal weight (<85th percentile), overweight or obese (≥ 85th percentile), and obese (≥ 95th percentile).

### Independent variables

#### Family income

As an indicator of economic resources, we used the child’s eligibility for the NSLP free or reduced-price meal, which is based on having a family income of less than or equal to 185% of the poverty level (18). NSLP is a commonly used indicator of family income (10,19) and was obtained from school enrollment forms. NSLP eligibility, as a dichotomous variable, was our main independent variable.

#### Confounders and effect measure modifiers

Other variables included in these analyses were age (continuous, calculated from birth date), sex, and race/ethnicity, which were originally derived from the enrollment records. Parents were asked to identify their child’s race or ethnicity from 1 of the following 8 categories: American Indian or Alaska Native, Asian, Pacific Islander or Native Hawaiian, Filipino, Hispanic or Latino, African American, white, and 2 or more races.

Because the literature suggests that the relationship between SES and overweight or obesity may vary by sex, age, and race/ethnicity, we explored whether each of these were effect measure modifiers (variables by which the association of exposure [family income] on the outcome [dependent variables] might vary). We used a Directed Acyclic Graph to identify hypothesized confounders (20). Physical fitness was not included in the models of BMI *z*-score/obesity because it is most likely a causal intermediate rather than a confounder. We planned to treat age, sex, and race/ethnicity as confounders if they were not found to be effect measure modifiers.

### Statistical analyses

Statistical analyses were performed using Stata version 12. Multiple linear regression models tested the association between family income and the continuous outcomes (fitness score and BMI *z*-score, separately). Log-binomial regression models (chosen because obesity is a common outcome, in which case the odds ratio overestimates the risk ratio [21]) examined the relative risk (RR) of obesity, comparing children with lower family income (NSLP eligible) to those with higher family income (NSLP ineligible).

In our statistical model building, we visually checked residual plots, which did not indicate any violations of the functional form. We first tested whether race/ethnicity, sex, and age were effect measure modifiers of the relationship between family income and each of our dependent variables (fitness score, BMI *z*-score, and obesity) by including all 3 interaction terms in a “full model” and testing the significance of each interaction term against the full model (22). All interactions were significant. Therefore, all models were stratified by race/ethnicity and sex. Within the sex- and race/ethnicity–specific models, age and age-by-family-income interactions were also included. Cluster-robust standard errors accounted for clustering of children within school districts and allowed for heteroskedasticity.

## Results

Children’s mean age was 13.0 (standard deviation [SD], 1.70) years. Mean BMI *z*-score was 0.63 (SD, 1.10); the prevalence of obesity was 20.3% (Table 1)

**Table 1:** Demographic Characteristics of Children, by Family Income Level, California, 2010–2012

Characteristic	Low Family Income	High Family Income	Total
**No. (%)**	901,463 (56)	715,937 (44)	1,617,400 (100)
**Sex, no. (%)**			
Boys	460,647 (56)	364,048 (44)	824,695 (100)
Girls	440,816 (56)	351,889 (44)	792,705 (100)
**Age, mean (SD), y**	12.9 (1.70)	13.1 (1.70)	13.0 (1.70)
**Race/ethnicity, no. (%)**			
American Indian or Alaska Native	5,838 (54)	4,931 (46)	10,769 (100)
Asian	47,018 (35)	87,601 (65)	134,619 (100)
Pacific Islander or Native Hawaiian	5,181 (57)	3,915 (43)	9,096 (100)
Filipino	15,909 (34)	30,316 (66)	46,225 (100)
Hispanic or Latino	641,768 (75)	208,547 (25)	850,315 (100)
African American	68,032 (67)	34,269 (33)	102,301 (100)
White	101,036 (24)	316,662 (76)	417,698 (100)
2 or more races/ethnicities	16,681 (36)	29,696 (64)	46,377 (100)
**Height, mean (SD), cm**	155.6 (11.43)	157.7 (11.86)	156.5 (11.66)
**Weight, mean (SD), kg**	55.5 (17.39)	53.0 (15.87)	54.4 (16.78)
**BMI z-score,^a^ mean (SD)**	0.80 (1.09)	0.43 (1.08)	0.63 (1.10)
**Physical fitness score,^b^ mean (SD)**	4.20 (1.51)	4.77 (1.35)	4.45 (1.47)
**Prevalence of obesity,^c^ %**	25.3	14.0	20.3

Abbreviations: BMI, body mass index; SD, standard deviation.

a BMI z-score based on 2000 Centers for Disease Control and Prevention Growth Reference in the United States.

b Physical fitness score ranged from 0 (least healthy) to 6 (most healthy).

c Obesity defined as a BMI percentile greater than or equal to 95th percentile based on CDC 2000 BMI-for-age growth reference.

A total of 56% of children were eligible for the NSLP. Compared with their representation in the total population, Hispanic or Latino and African American children were disproportionately represented among children with lower family income. Asian and white children were disproportionately represented in the higher income category. Unadjusted average weight of children with lower family income was approximately 2.5 kg higher than that of higher income children; BMI *z*-score was 0.37 units higher, and obesity prevalence was nearly twice as high among children with lower versus higher income (Table 1). Unadjusted fitness scores were also significantly lower (indicating less fitness) among the children with lower (vs higher) family income.

### Associations between family income and physical fitness

The race/ethnicity- and sex-stratified and age-adjusted difference in mean fitness score, comparing children with higher family income to those with lower family income at mean age, was significant in all racial/ethnic groups and in both sexes. The magnitude was the largest for Pacific Islander or Native Hawaiians among both boys (−0.49; 95% CI, −0.59 to −0.39) and girls (−0.62; 95% CI, −0.71 to −0.52) (Table 2). Among boys, American Indian or Alaska Natives (−0.41; 95% CI, −0.49 to −0.32) and whites (−0.43; 95% CI, −0.46 to −0.40) also had larger differences in fitness score between children with lower versus higher family income at mean age than those of other racial/ethnic groups. For girls, the difference was larger for whites (−0.60; 95% CI, −0.64 to −0.56), children of 2 or more races (−0.55; 95% CI, −0.62 to −0.47), and American Indian or Alaska Natives (−0.52; 95% CI, −0.62 to −0.42) compared with other racial/ethnic groups (Table 2).

**Table 2:** Difference in Fitness Score and BMI *z*-score Between Children With Lower Family Income and Children With Higher Family Income, by Race/Ethnicity at Mean Age, California, 2010–2012

Characteristic	Boys		Girls	
	Coefficient^a^ (95% CI)	*P* Value	Coefficient^a^ (95% CI)	*P* Value
**Fitness Score^b^**				
American Indian or Alaska Native	−0.41 (−0.49 to −0.32)	<.001	−0.52 (−0.62 to −0.42)	<.001
Asian	−0.29 (−0.34 to −0.24)	<.001	−0.39 (−0.45 to −0.33)	<.001
Pacific Islander or Native Hawaiian	−0.49 (−0.59 to −0.39)	<.001	−0.62 (−0.71 to −0.52)	<.001
Filipino	−0.19 (−0.23 to −0.15)	<.001	−0.25 (−0.30 to −0.19)	<.001
Hispanic or Latino	−0.26 (−0.29 to −0.22)	<.001	−0.33 (−0.37 to −0.29)	<.001
African American	−0.21 (−0.25 to −0.17)	<.001	−0.37 (−0.41 to −0.32)	<.001
White	−0.43 (−0.46 to −0.40)	<.001	−0.60 (−0.64 to −0.56)	<.001
2 or more races/ethnicities	−0.30 (−0.35 to −0.24)	<.001	−0.55 (−0.62 to −0.47)	<.001
**BMI z-score^c^**				
American Indian or Alaska Native	0.25 (0.18 to 0.32)	<.001	0.26 (0.20 to 0.31)	<.001
Asian	0.18 (0.13 to 0.22)	<.001	0.20 (0.16 to 0.24)	<.001
Pacific Islander or Native Hawaiian	0.41 (0.33 to 0.49)	<.001	0.40 (0.33 to 0.48)	<.001
Filipino	0.09 (0.05 to 0.13)	<.001	0.10 (0.05 to 0.14)	<.001
Hispanic or Latino	0.17 (0.15 to 0.19)	<.001	0.16 (0.14 to 0.18)	<.001
African American	0.09 (0.07 to 0.11)	<.001	0.16 (0.13 to 0.18)	<.001
White	0.27 (0.25 to 0.29)	<.001	0.32 (0.30 to 0.34)	<.001
2 or more races/ethnicities	0.20 (0.16 to 0.24)	<.001	0.33 (0.28 to 0.38)	<.001

Abbreviations: BMI, body mass index; CI, confidence interval.

a Coefficient from multiple linear regression model adjusting for age.

b Fitness score ranged from 0 (least healthy) to 6 (most healthy).

c BMI z-score determined based on 2000 Centers for Disease Control and Prevention Growth Reference in the United States.

### Association between family income and BMI z-score

The adjusted BMI *z*-score of lower income children was significantly higher than that of higher income children at mean age in both sexes and in all racial/ethnic groups. Both Pacific Islander or Native Hawaiian boys (0.41; 95% CI, 0.33–0.49) and girls (0.40; 95% CI, 0.33–0.48) had the highest difference in BMI z-score for children with lower versus higher family income at mean age. African American (0.09; 95% CI, 0.07–0.11) and Filipino (0.09; 95% CI, 0.05–0.13) boys had a smaller difference in BMI z-score than did other racial/ethnic groups. Among girls, the difference in mean BMI z-score was the smallest for Filipino (0.10; 95% CI, 0.05–0.14) (Table 2).

### Association between family income and obesity

The prevalence of obesity for both boys and girls with lower family income was significantly higher than for those with higher income at mean age among all racial/ethnic groups (Table 3). The largest magnitude of increased risk was seen among lower income white boys, who had 71% higher prevalence of obesity than higher income white boys (RR = 1.71; 95% CI, 1.64–1.79) at mean age. Filipino boys had only 17% and African American boys, only16% higher adjusted prevalence than their higher income counterparts. Among girls, lower-income white and Asian girls had 2.06 (95% CI, 1.95–2.17) and 2.00 (95% CI, 1.74–2.29) times higher prevalence of obesity than their higher income counterparts; Filipino, Hispanic or Latino, and African American girls with lower family income had only about 30% higher adjusted prevalence of obesity than their higher income counterparts (Filipino girls’ RR = 1.30 [95% CI, 1.17–1.44]; Hispanic or Latino girls’ RR = 1.29 [95% CI, 1.25–1.34]; and African American girls’ RR = 1.32 95% CI [1.26–1.39]) (Table 3).

**Table 3:** Relative Risk (RR) of Obesity of Children With Lower Family Income Compared With Children With Higher Family Income, by Race/Ethnicity at Mean Age, California, 2010–2012

Race/ethnicity	Obesity^a^			
	Boys		Girls	
	RR^b^ (95% CI)	*P* Value	RR^b^ (95% CI)	*P* Value
American Indian or Alaska Native	1.45 (1.30–1.63)	<.001	1.57 (1.41–1.74)	<.001
Asian	1.58 (1.45–1.72)	<.001	2.00 (1.74–2.29)	<.001
Pacific Islander or Native Hawaiian	1.60 (1.44–1.77)	<.001	1.76 (1.52–2.04)	<.001
Filipino	1.17 (1.10–1.24)	<.001	1.30 (1.17–1.44)	<.001
Hispanic or Latino	1.22 (1.19–1.26)	<.001	1.29 (1.25–1.34)	<.001
African American	1.16 (1.11–1.22)	<.001	1.32 (1.26–1.39)	<.001
White	1.71 (1.64–1.79)	<.001	2.06 (1.95–2.17)	<.001
Two or More races/ethnicities	1.38 (1.29–1.48)	<.001	1.82 (1.66–2.00)	<.001

Abbreviation: CI, confidence interval.

a Obesity defined as children’s BMI percentile greater than or equal to 95th percentile based on Centers for Disease Control and Prevention 2000 BMI-for-age growth reference.

b RR from log-binomial model adjusting for age.

## Discussion

We used data from approximately 1.6 million children from California from 2010 through 2012 to assess the association between family income and physical fitness score, BMI *z*-score, and obesity. For all 8 racial/ethnic groups, children with lower family income were found to be less physically fit, have higher BMI *z*-score, and have higher prevalence of obesity than children with higher family income. The percentage of children with low family income was disproportionately high among Hispanic or Latino and African American children. Approximately 75% of Hispanic or Latino and 67% of African American children were identified as low income, compared with only 24% of white children.

Previous studies of children’s physical fitness have been limited to examining either income or race/ethnicity and its relationship to physical fitness. Two previous studies also used the Fitnessgram protocol to examine children’s fitness and also reported higher physical fitness for children with higher income, but no race-specific relationships were tested in these studies (19,23). A separate study examined racial/ethnic differences in physical activity and found that white and Asian children were more likely to participate in moderate to vigorous activity than Hispanic and African American children (24). We expand on this work by showing that children with lower family income were found to be significantly less physically fit compared with higher income children in 8 different racial/ethnic groups. In our study, the magnitude of the association between family income and fitness scores was smaller for Filipino, Hispanic or Latino, and African American children than that seen in American Indian or Alaska Native and white children.

There are several possible reasons for the relationship between low family income and lower physical fitness. First, populations with low SES may be more likely to live in low SES communities. Parents from low SES communities have reported higher perceived neighborhood crime and unpleasant neighborhood features compared with those from high SES communities. This concern for safety could make low SES parents less inclined to let their children play outside, thereby restricting physical activity (25). Low SES communities may have fewer physical activity facilities, particularly few free activity resources, than high SES communities, thus limiting access to opportunities for physical activity (7). At an individual level, lower income families may experience many stressors and barriers to health and as a result may place less priority on leisure time physical activity compared with higher income families (26). Many leisure activities may also be cost-prohibitive for low-income parents due to membership, participation, or equipment fees (7).

Within all racial/ethnic groups, children with lower family income also had significantly higher risk of obesity than children with higher family income. This finding contrasts with those of previous studies, which have shown that SES is inversely related to obesity only among white children (27,28). In fact, the magnitude of the association between family income and obesity was as large for Asian girls as it was for white girls and was only slightly higher than the magnitude seen among girls of 2 or more races and Pacific Islanders or Native Hawaiians. Similar findings were seen among boys, where whites also had the largest magnitude of association, but this was only slightly higher than that seen for Asians and Pacific Islanders or Native Hawaiians. These findings are in contrast to findings from Hispanic and African American children in NHANES. Previous NHANES data showed no consistent pattern for Hispanic children, and among African American children, there was a positive association (27). In another study of teens and young adults, only white children (and not black or Hispanic children) showed clear decreasing overweight prevalence with increasing income (28). We suspect that our large sample size allowed us to detect these differences within racial/ethnic groups and sex, whereas the limited nonwhite sample in NHANES is not able to pick up these differences. However, the magnitude of increased risk was substantial and cannot be a case of picking up very small differences as merely a result of having a large sample. By pooling over a range of ages, analyses using NHANES data may have missed these disparities in children aged 10 to 17 years.

Potential reasons for this association between family income and obesity are the same as those for the association with physical activity, because low physical activity participation and sedentary lifestyle could be the cause of higher prevalence of overweight and obesity (25). Additional reasons for the observed relationship could include the price of healthy food compared with unhealthy food and food insecurity (29).

In terms of sex differences and the association between family income and fitness score, within racial/ethnic groups, income-based differences were larger for girls than boys, in general. Boys are generally more likely to participate in physical activity than girls (30), so boys with low family income could be more physically active than their female counterparts, resulting in less difference in fitness scores between lower family and higher family income.

A strength of this study was the large sample size and diverse population, which allowed us to assess whether family income was associated with obesity and fitness for 8 racial/ethnic groups. An additional strength was the measurement of fitness, rather than using self-reported activity. Finally, the data are representative of public school students in California.

Our study also has limitations. Data from students in private schools were not available. Use of data from this population would influence the estimation of actual sociodemographic distribution of the students in California, because private school students may have different socioeconomic characteristics, including higher incomes. NSLP information was not available in the data before 2010, so we were unable to analyze time trends in these relationships. Family income was only categorized into 2 groups on the basis of NSLP eligibility, which may have limited power because there is less distinction between the highest and the lowest income groups. Area SES may influence the physical activity of children but was not analyzed in this study (7,25). Also, we cannot determine whether the association between family income and childhood obesity or children’s physical fitness is causal because the data used were cross-sectional. We did not have detailed information on additional parental characteristics, such as education, occupation, BMI, or motivation, all of which could be associated with family income and may also be associated with child BMI and physical fitness, creating unmeasured confounding (31). Finally, the results may not be generalizable to other states.

We conclude that children with low family income tend to be less physically fit and have higher risk of obesity than children with higher family income. We observed this relationship for 8 racial/ethnic groups and for both boys and girls. This information is relevant for targeting policies and programs aimed at improving the fitness levels and decreasing the obesity risk of children.
